# Dipyridamole combined with immunoglobulin and aspirin in the treatment of Kawasaki disease in children: a meta-analysis

**DOI:** 10.3389/fmed.2026.1837742

**Published:** 2026-06-29

**Authors:** Yanshuo Shi, Xin Xu, Yuanyuan Yue, Jianqun Zhao, Kaiqing Yao, Huizhen Wu

**Affiliations:** 1 Department of Pharmacy, Hebei General Hospital, Shijiazhuang, China; 2 Hebei Key Laboratory of Clinical Pharmacy, Shijiazhuang, China; 3 Department of Dressing, Hebei General Hospital, Shijiazhuang, China; 4 Gaocheng Vocational Technology Education Center, Shijiazhuang, China

**Keywords:** aspirin, dipyridamole, immunoglobulin, Kawasaki disease, meta-analysis

## Abstract

**Objective:**

The objective of this study was to systematically evaluate the clinical efficacy and safety of dipyridamole combined with immunoglobulin and aspirin in the treatment of Kawasaki disease (KD) in children.

**Methods:**

A computerized search was conducted in databases including PubMed, China Biology Medicine disc (CBM), China National Knowledge Infrastructure (CNKI), Wanfang Data Knowledge Service Platform (Wanfang), and VIP Chinese Sci-Tech Journals Full-text Database (VIP), covering the period from the inception of each database to February 2026. We collected randomized controlled trials (RCTs) that compared the clinical efficacy and safety of dipyridamole combined with immunoglobulin and aspirin (observation group) agains immunoglobulin combined with aspirin (control group) in the treatment of KD in children, in conjunction with standard therapy. After data extraction and quality assessment of clinical studies meeting the inclusion criteria, a meta-analysis was performed using RevMan 5.3 statistical software.

**Results:**

A total of 18 RCTs involving 1,594 patients were included. The meta-analysis results indicated that the total effective rate in the observation group was significantly higher than that in the control group. Additionally, the time for clinical symptom improvement, C-reactive protein levels (CRP), erythrocyte sedimentation rate (ESR), platelet count (PLT), coronary artery lesion (CAL) and fibrinogen levels (FIB) were all significantly lower in the observation group compared to the control group. There was no significant difference in the incidence of adverse reactions between the two groups.

**Conclusion:**

The clinical efficacy of dipyridamole combined with immunoglobulin and aspirin in treating Kawasaki disease in children is superior to that of immunoglobulin combined with aspirin, with comparable safety.

**Systematic review registration:**

https://www.crd.york.ac.uk/PROSPERO/view/CRD420261336468, identifier CRD420261336468.

## 1 Introduction

Kawasaki disease (KD) is an acute pediatric vasculitis characterized by high fever, rash, mucocutaneous inflammation, and cervical lymphadenopathy, with its etiology remaining unclear (1, 2). The disease progresses rapidly and may lead to irreversible coronary artery damage if not treated promptly. The primary clinical treatment involves early high-dose intravenous immunoglobulin (IVIG) therapy combined with aspirin to suppress coronary inflammation and thrombosis (3). However, approximately 10% of patients exhibit IVIG resistance or aspirin intolerance, resulting in suboptimal therapeutic outcomes (4). Current clinical studies have found that dipyridamole exhibits antiplatelet, anti-inflammatory, and coronary vasodilating effects, demonstrating favorable clinical efficacy against abnormal platelet aggregation, vascular endothelial inflammatory responses, and coronary blood flow obstruction (5). While there is an increasing number of clinical reports on the combined use of dipyridamole with immunoglobulin and aspirin for treating KD in children, individual studies have small sample sizes and lack systematic evidence-based medical support. Therefore, this study systematically evaluates the efficacy and safety of dipyridamole combined with immunoglobulin and aspirin versus immunoglobulin combined with aspirin in the treatment of KD in children. We aim to provide evidence-based guidance for rational clinical medication selection.

## 2 Materials and methods

### 2.1 Inclusion and exclusion criteria

#### 2.1.1 Diagnostic criteria

The diagnostic criteria were based on the KD diagnostic criteria established by the American Heart Association (6).

#### 2.1.2 Study type

This study includes randomized controlled trials (RCTs) on the treatment of KD with dipyridamole combined with immunoglobulin and aspirin, irrespective of language.

#### 2.1.3 Study subjects

The patients we selected meet the diagnostic criteria for Kawasaki Disease (KD). They have not received any relevant treatment in the past, and their guardians have signed informed consent forms. Additionally, the patients are under 8 years of age.

#### 2.1.4 Intervention measures

Patients in the control group were treated with a combination of immunoglobulin and aspirin, while the observation group received dipyridamole in addition to immunoglobulin and aspirin. In the control group, immunoglobulin was administered at a dosage of 2 g/(kg·d) via slow infusion over a period of 8 to 12 h, initiated within 10 days of the disease onset. The initial dosage of aspirin was 25 to 50 mg/(kg·d), which was subsequently reduced to 3 to 5 mg/(kg·d) once the patients’ body temperature returned to normal. The observation group received an additional dipyridamole dosage of 5 mg/(kg·d) based on the treatment regimen of the control group. The treatment duration for both groups was 6 to 8 weeks.

#### 2.1.5 Outcome indicators

The primary outcome indicators include: a. Clinical efficacy, defined as the total effective rate, calculated by the formula: Total effective rate = (Number of cured cases + markedly improved cases + improved cases) / Total number of cases (7); b. The time taken for the improvement of related clinical symptoms, which include fever, limb swelling, conjunctival congestion, trunk erythema, lymphadenopathy, and diffuse mucosal congestion (8); c. Relevant laboratory indicators, which encompass CRP, ESR, PLT, CAL and FIB (9); d. The incidence of adverse reactions, which serves as a safety evaluation indicator.

In the context of RCTs, efficacy assessment criteria can be categorized as follows: Cured: Following treatment, the child’s symptoms, including fever, swelling of the hands and feet, and mucosal congestion, have resolved completely, and the complete blood count results indicate normal levels. Markedly Improved: After treatment, the child’s symptoms have significantly diminished; however, the complete blood count still reveals one abnormal indicator. Improved: Post-treatment, the child’s condition has shown improvement, with alleviated symptoms; nonetheless, the complete blood count has not yet returned to normal.

In the RCTs included in this study, the evaluation of CAL was conducted following a treatment period of 2 months. The Z-score obtained from echocardiography served as the diagnostic criterion for CAL, with a threshold of Z-score ≥ 2.5.

#### 2.1.6 Exclusion criteria

Non-RCT; Duplicate publications; Review articles, conference papers; Sample size <20; Patients who had received KD treatment prior to hospitalization; Literature with unavailable full-text data.

### 2.2 Search strategy

Computerized searches were conducted in PubMed, CNKI, Wanfang, CBM, and VIP databases (from their inception to February 2026). The search terms included: “Dipyridamole,” “Immunoglobulin,” “Aspirin,” “KD,” “Kawasaki disease,” “Mucocutaneous lymph node syndrome”, “Random.”

### 2.3 Data extraction and quality assessment

Two researchers independently conducted the literature screening to determine the inclusion of studies for this research. The extracted information encompassed the title, authors, subjects, methods, interventions, outcomes, blinding, and allocation concealment. The studies were evaluated by the two researchers using the Cochrane risk of bias assessment tool (10), which assessed the following aspects: random sequence generation, allocation concealment, blinding, incomplete outcome data, and other biases. The methodological quality was independently assessed by two reviewers, with any disagreements resolved through discussion or by consulting a third reviewer.

### 2.4 Statistical methods

Statistical analysis was conducted using RevMan 5.3 software. For dichotomous data, relative risk (RR) with a 95% confidence interval (CI) was utilized. A fixed-effect model was applied when no statistical heterogeneity was observed among studies (*I*^2^ ≤ 50%, *p* ≥ 0.01). Conversely, a random-effects model was employed in the presence of statistical heterogeneity (*I*^2^ > 50%, *p* < 0.01) (11). For continuous variables, the mean difference (MD) served as the effect measure. Forest plots were utilized to present the analytical results of all data, while funnel plots were used to evaluate publication bias (12). A *p*-value of less than 0.05 was deemed statistically significant.

## 3 Results

### 3.1 Basic information of the included studies

**Figure 1:**
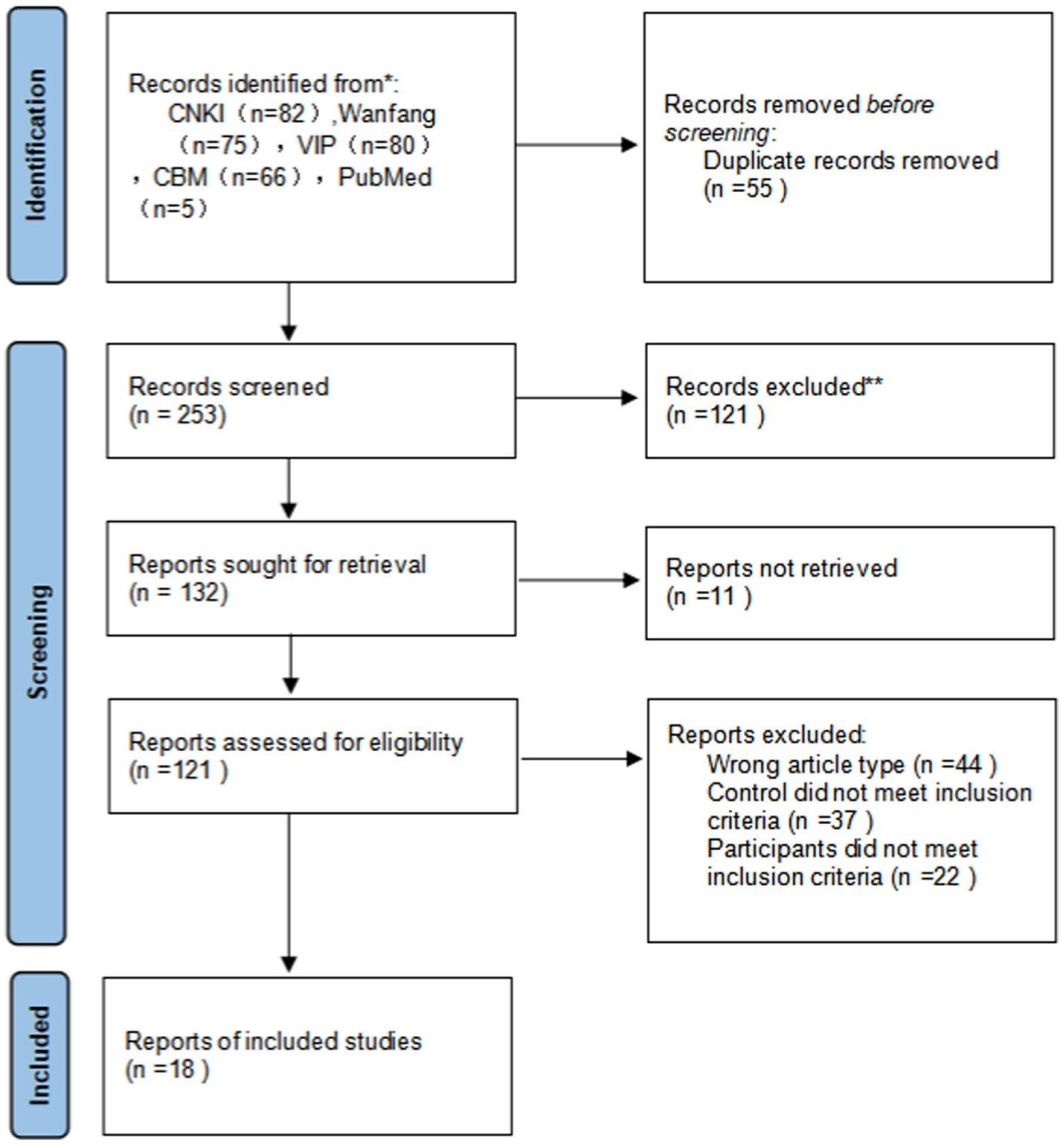
PRISMA flowchart of literature search.

Due to the limited clinical data on the use of dipyridamole combined with immunoglobulin in the treatment of KD internationally, all 18 RCTs included in this study were derived from Chinese literature (13–30). A total of 1,594 cases were analyzed, comprising 799 in the observation group and 795 in the control group, with the screening process illustrated in Figure 1. The minimum sample size in the observation group was 30 cases, while the maximum was 75 cases. The observation group received a combination of dipyridamole, immunoglobulin, and aspirin, along with basic treatment, whereas the control group was treated with immunoglobulin, aspirin, and basic treatment. The fundamental characteristics of the included studies are summarized in Table 1.

**Table 1:** General information of included studies.

First author publication year	Sample size		Male/female		Age		Intervening measure		Outcome indicators
	T	C	T	C	T	C	T	C	
Feng et al. 2022 (13)	49	47	18/31	19/28	3.34 ± 0.75	3.52 ± 0.52	Dipyridamole + Immunoglobulin + Aspirin	Immunoglobulin + Aspirin	b,f,g,h
Li et al. 2022 (14)	34	34	16/18	17/17	2.73 ± 0.49	2.79 ± 0.62	Dipyridamole + Immunoglobulin + Aspirin	Immunoglobulin + Aspirin	b
Li et al. 2022 (15)	40	40	23/17	21/19	2.58 ± 0.68	2.49 ± 0.68	Dipyridamole + Immunoglobulin + Aspirin	Immunoglobulin + Aspirin	a,c,f,h
Yang et al. 2020 (16)	37	37	23/14	22/15	2.86 ± 0.63	2.84 ± 0.62	Dipyridamole + Immunoglobulin + Aspirin	Immunoglobulin + Aspirin	b,c,h
Lin et al. 2019 (17)	46	45	25/21	23/22	2.47 ± 0.82	2.54 ± 0.81	Dipyridamole + Immunoglobulin + Aspirin	Immunoglobulin + Aspirin	a,b,c,h
Lin et al. 2022 (18)	41	40	22/19	23/17	3.0 ± 0.9	2.9 ± 0.8	Dipyridamole + Immunoglobulin + Aspirin	Immunoglobulin + Aspirin	b,c
Wu et al. 2023 (19)	40	40	22/18	23/17	5.81 ± 2.03	6.51 ± 2.75	Dipyridamole + Immunoglobulin + Aspirin	Immunoglobulin + Aspirin	a,b,c,g,h
Fu et al. 2022 (20)	36	36	19/17	18/18	3.62 ± 2.56	3.71 ± 2.63	Dipyridamole + Immunoglobulin + Aspirin	Immunoglobulin + Aspirin	a,b
Jiao et al. 2016 (21)	59	59	28/31	29/30	2.62 ± 0.61	2.85 ± 0.96	Dipyridamole + Immunoglobulin + Aspirin	Immunoglobulin + Aspirin	a,b,c,h
Wang et al. 2021 (22)	75	75	34/41	36/39	3.45 ± 0.86	3.71 ± 0.82	Dipyridamole + Immunoglobulin + Aspirin	Immunoglobulin + Aspirin	b,f,g
Wang et al. 2021 (23)	47	47	29/18	25/22	7.0 ± 6.0	7.0 ± 6.0	Dipyridamole + Immunoglobulin + Aspirin	Immunoglobulin + Aspirin	a,b,h
Wang et al. 2023 (24)	30	30	14/16	16/14	1.75 ± 1.08	1.93 ± 1.43	Dipyridamole + Immunoglobulin + Aspirin	Immunoglobulin + Aspirin	a,b,c,d,e,f,g
Wang et al. 2023 (25)	43	43	26/17	27/16	2.54 ± 0.79	2.47 ± 0.82	Dipyridamole + Immunoglobulin + Aspirin	Immunoglobulin + Aspirin	a,b,c,d,e,f,g,h
Wang et al. 2022 (26)	60	60	33/27	34/26	3.56 ± 1.16	3.89 ± 1.63	Dipyridamole + Immunoglobulin + Aspirin	Immunoglobulin + Aspirin	a,c,d,e,h
Shao et al. 2022 (27)	48	48	29/19	32/16	6.85 ± 1.28	7.75 ± 1.17	Dipyridamole + Immunoglobulin + Aspirin	Immunoglobulin + Aspirin	a,c
Zou et al. 2018 (28)	30	30	17/13	19/11	2.92 ± 0.11	3.24 ± 0.23	Dipyridamole + Immunoglobulin + Aspirin	Immunoglobulin + Aspirin	a,b,d,e,g
Guo et al. 2022 (29)	41	41	27/14	28/13	3.1 ± 1.1	3.4 ± 0.9	Dipyridamole + Immunoglobulin + Aspirin	Immunoglobulin + Aspirin	a,f
Ma et al. 2023 (30)	43	43	24/19	26/17	3.89 ± 1.13	3.86 ± 0.97	Dipyridamole + Immunoglobulin + Aspirin	Immunoglobulin + Aspirin	a,f

T:experimental group; C:control group; a. Effective rate; b. Time to clinical symptom improvement; c. CRP; d. ESR; e. PLT; f. FIB; g. CAL; h.adverse effect.

### 3.2 Evaluation of methodological quality and risk assessment of included studies

**Figure 2:**
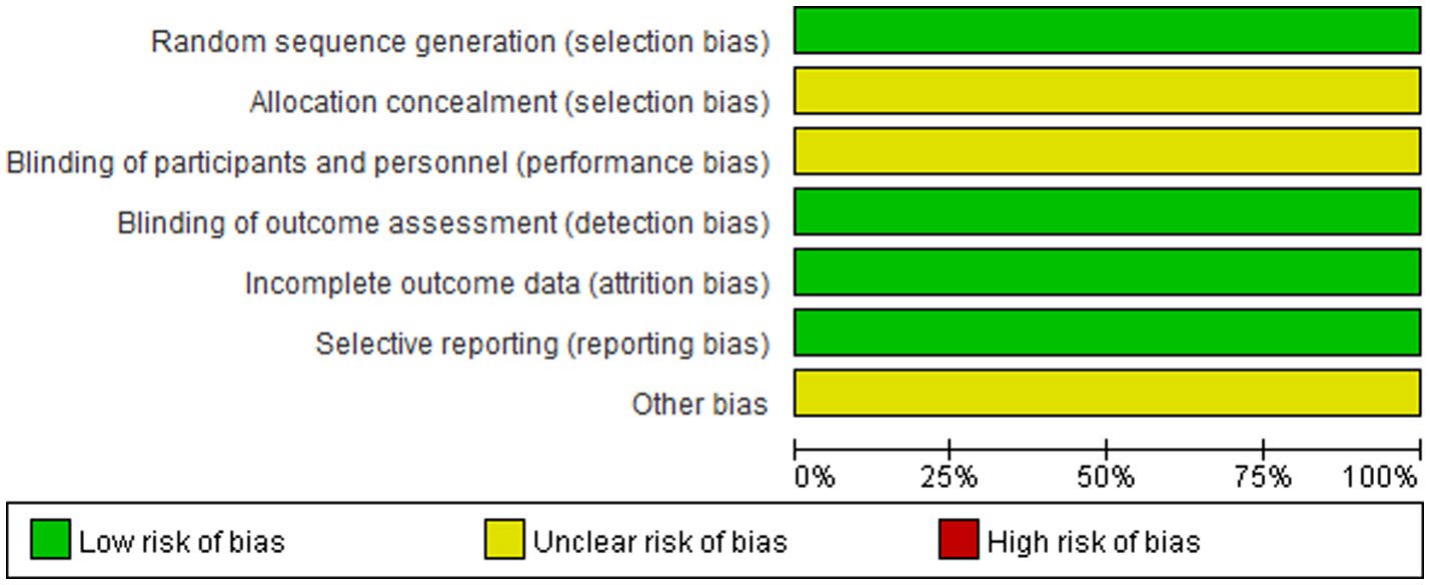
Bias risk assessment of included literature.

Among the 18 RCTs analyzed, 15 utilized random number tables (13–17, 19–27, 30), while 3 employed random grouping methods (18, 28, 29). All studies reported complete data with no instances of loss to follow-up or attrition. A detailed methodological quality assessment of the included studies is provided in Table 2. The risk of bias for these studies was evaluated using RevMan 5.3 software, and the results of this assessment are illustrated in Figure 2. The Cochrane risk-of-bias table with judgments for each domain are provided in Supplementary material.

**Table 2:** Methodological quality evaluation.

Literature resources random grouping method blind method shedding case literature quality	Literature resources random grouping method blind method shedding case literature quality	Literature resources random grouping method blind method shedding case literature quality	Literature resources random grouping method blind method shedding case literature quality	Literature resources random grouping method blind method shedding case literature quality
Feng et al. 2022 (13)	Random number table	Not mentioned	Null	B
Li et al. 2022 (14)	Random number table	Not mentioned	Null	B
Li et al. 2022 (15)	Random number table	Not mentioned	Null	B
Yang et al. 2020 (16)	Random number table	Not mentioned	Null	B
Lin et al. 2019 (17)	Random number table	Not mentioned	Null	B
Lin et al. 2022 (18)	random grouping	Not mentioned	Null	B
Wu et al. 2023 (19)	Random number table	Not mentioned	Null	B
Fu et al. 2022 (20)	Random number table	Not mentioned	Null	B
Jiao et al. 2016 (21)	Random number table	Not mentioned	Null	B
Wang et al. 2021 (22)	Random number table	Not mentioned	Null	B
Wang et al. 2021 (23)	Random number table	Not mentioned	Null	B
Wang et al. 2023 (24)	Random number table	Not mentioned	Null	B
Wang et al. 2023 (25)	Random number table	Not mentioned	Null	B
Wang et al. 2022 (26)	Random number table	Not mentioned	Null	B
Shao et al. 2022 (27)	Random number table	Not mentioned	Null	B
Zou et al. 2018 (28)	random grouping	Not mentioned	Null	B
Guo et al. 2022 (29)	random grouping	Not mentioned	Null	B
Ma et al. 2023 (30)	Random number table	Not mentioned	Null	B

### 3.3 Meta-analysis results

#### 3.3.1 The total effective rate

**Figure 3:**
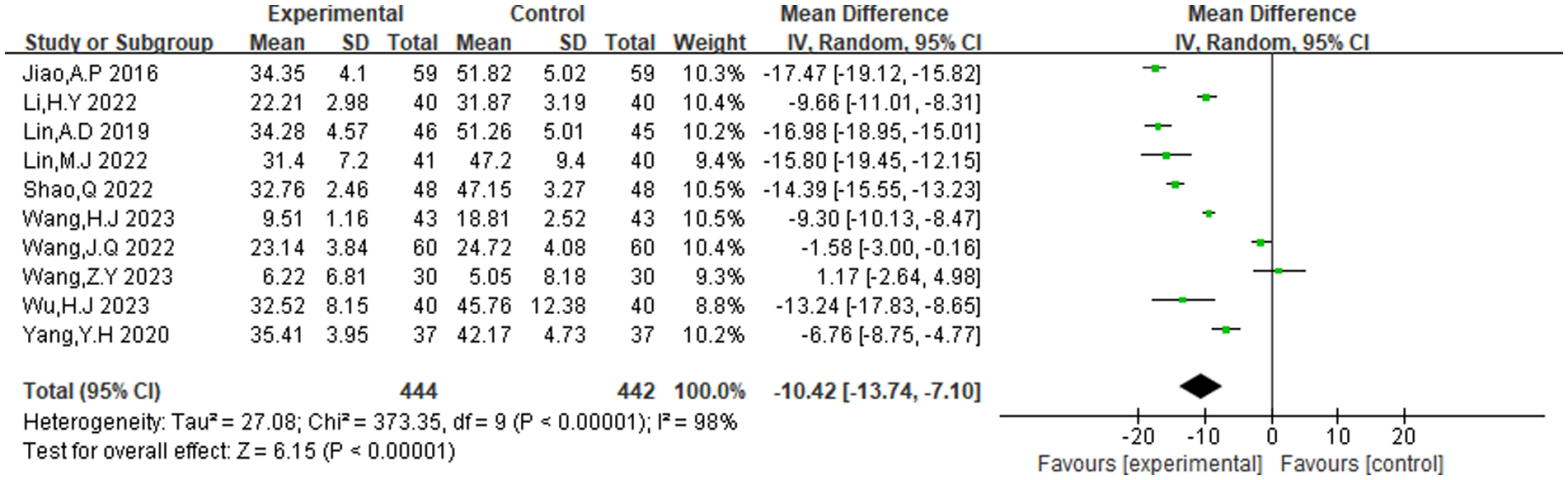
Meta-analysis of the total effective rate in studies.

A total of 13 studies (15, 17, 19–21, 23–30) reported total effective rate. After conducting a heterogeneity test (*p* = 0.52, *I*^2^ = 0%), a fixed-effects model was employed. The systematic evaluation results indicated that the overall response rate in the observation group was significantly higher than that in the control group, showing a statistically significant difference (RR = 1.19, 95% CI [1.13, 1.24], *p* < 0.00001), as illustrated in Figure 3.

#### 3.3.2 Improvement time of clinical symptoms

A total of 13 studies (13, 14, 16–25, 28) reported the improvement time of clinical symptoms, with primary indicators including fever, limb swelling, conjunctival congestion, trunk erythema, lymphadenopathy, and diffuse mucosal congestion. Subgroup analyses were conducted for each indicator, and the results are presented in Table 3. As shown in the table, the observation group exhibited significantly shorter improvement times for all types of clinical symptoms compared to the control group, with statistically significant differences.

**Table 3:** Meta-analysis of time to clinical symptom improvement.

Category	Number of publications	Heterogeneity		RR(95%CI)	*p*
		*p*	*I* ^2^		
Fever	13 (13, 14, 16–25, 28)	<0.00001	78%	−1.18(−1.35, −1.01)	<0.00001
Limb swelling	4 (20, 23, 24, 28)	<0.00001	95%	−2.46(−2.71, −2.20)	<0.00001
Conjunctival congestion	7 (13, 14, 16–18, 21, 22)	=0.07	48%	−0. 85(−1.01, −0.68)	<0.00001
Truncal erythema	10 (13, 14, 16, 17, 19–23, 25)	<0.00001	84%	−1.10(−1.38, −0.82)	<0.00001
Lymphadenopathy	11 (13, 14, 16–19, 21–23, 25, 28)	<0.00001	98%	−1.7 9(−2.57, −1.01)	<0.00001
Diffuse mucosal hyperemia	12 (13, 14, 17–25, 28)	<0.00001	82%	−1.83 (−2.14, −1.52)	<0.00001

To explore the sources of heterogeneity in the three clinical symptom indicators—fever, lymphadenopathy, and diffuse mucosal congestion—we conducted a sensitivity analysis (refer to the Supplementary file). The results indicate that the direction of the combined effect size remained unchanged following the sequential exclusion of each study, and the statistical significance was preserved (*p* < 0.0001), suggesting that the combined effect results are robust. However, the heterogeneity did not significantly decrease, which may be attributed to clinical or methodological differences between studies, such as the criteria for symptom assessment and the timing of measurements.

#### 3.3.3 CRP

**Figure 4:**
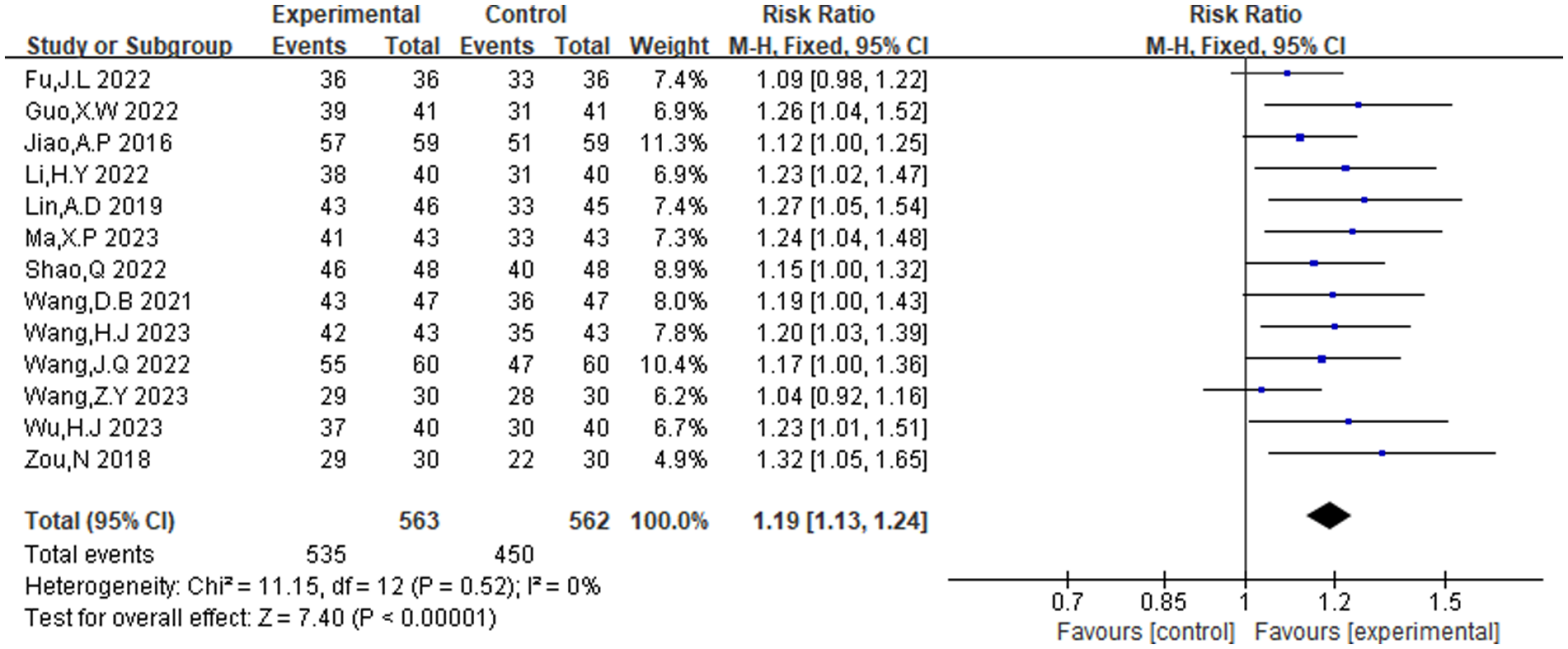
Meta-analysis of CRP in studies.

A total of 10 studies (15–19, 21, 24–27) reported CRP levels. After detecting heterogeneity (*p* < 0.00001, *I*^2^ = 98%), a random-effects model was adopted. The systematic review results demonstrated that the CRP levels in the observation group were significantly lower than those in the control group, with a statistically significant difference (MD = −10.42, 95% CI [−13.74, −7.10], *p* < 0.00001), as shown in Figure 4.

To explore the sources of high heterogeneity in CRP, we conducted a sensitivity analysis (see Supplementary file). By sequentially removing each study, we observed that the pooled effect size (MD) ranged from −9.61 to −11.60, with all results being statistically significant (p < 0.00001). This indicates the robustness of the findings, as no single study dominated the overall effect. However, the sensitivity analysis did not significantly reduce the heterogeneity, as the *I*^2^ statistic remained above 96%. This suggests that the observed heterogeneity may be attributed to clinical or methodological differences among the studies, including variations in baseline CRP levels, dosages of dipyridamole, and measurement time points.

#### 3.3.4 ESR

**Figure 5:**
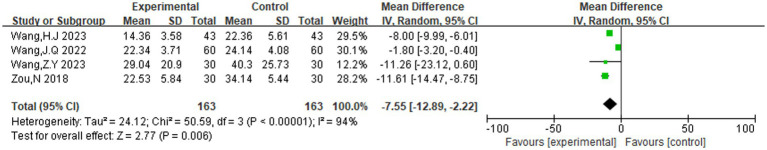
Meta-analysis of ESR in studies.

Four studies reported on the ESR (24–26, 28). After detecting significant heterogeneity (*p* < 0.00001, *I*^2^ = 94%), a random-effects model was employed. The results of the systematic review indicated that the ESR in the observation group was significantly lower than that in the control group, with a statistically significant difference (MD = −7.55, 95% CI [−12.89, −2.22], *p* = 0.006), as illustrated in Figure 5.

To explore the source of high heterogeneity in the ESR, we conducted a sensitivity analysis (see Supplementary file). By sequentially excluding each study, we observed that the exclusion of Wang et al. (26) led to a significant decrease in heterogeneity to 53% (*p* = 0.12), while the pooled effect size remained significant (MD = −9.70, 95%CI: −12.66 to −6.74, *p* < 0.00001). Conversely, excluding other studies did not result in a significant alteration of heterogeneity. Furthermore, after excluding Wang et al. (26), the pooled effect size lost its statistical significance (*p* = 0.07). These findings suggest that Wang et al. (26) may be a primary source of heterogeneity. An analysis of the differences between this study and the other included studies indicates that factors influencing heterogeneity may include baseline values, patient age, and gender differences. The robustness of the overall conclusion necessitates further validation.

#### 3.3.5 PLT

**Figure 6:**
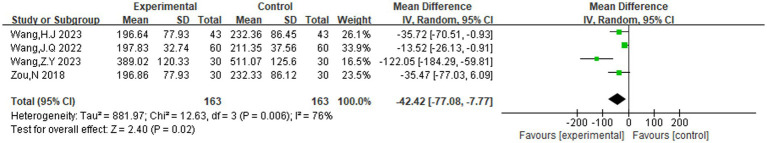
Meta-analysis of PLT in studies.

A total of 4 studies (24–26, 28) reported PLT levels. After detecting heterogeneity (*p* = 0.006, *I*^2^ = 76%), a random-effects model was adopted. The systematic review results demonstrated that the observation group had significantly lower PLT levels compared to the control group (MD = -42.42, 95% CI [−77.08, −7.77], *p* = 0.02), as shown in Figure 6.

To explore the sources of high heterogeneity, we conducted a sensitivity analysis (see Supplementary file). Sequential exclusion of each study revealed that the pooled effect size (MD) ranged from −18.61 to −58.04, consistently indicating that the dipyridamole group exhibited a superior effect in reducing PLT. Notably, the exclusion of Wang (24) led to a significant decrease in heterogeneity to 7% (*p* = 0.34), with the pooled effect size changing to −18.61 (95% CI: −31.70 to −5.51, *p* = 0.005). In contrast, the exclusion of other studies continued to yield high heterogeneity (*I*^2^ > 68%). Furthermore, excluding Wang et al. (25) resulted in the loss of statistical significance for the pooled effect size (*p* = 0.06). These findings suggest that Wang (24) may be a primary contributor to the observed heterogeneity, significantly influencing the magnitude of the overall effect size.

#### 3.3.6 FIB

**Figure 7:**
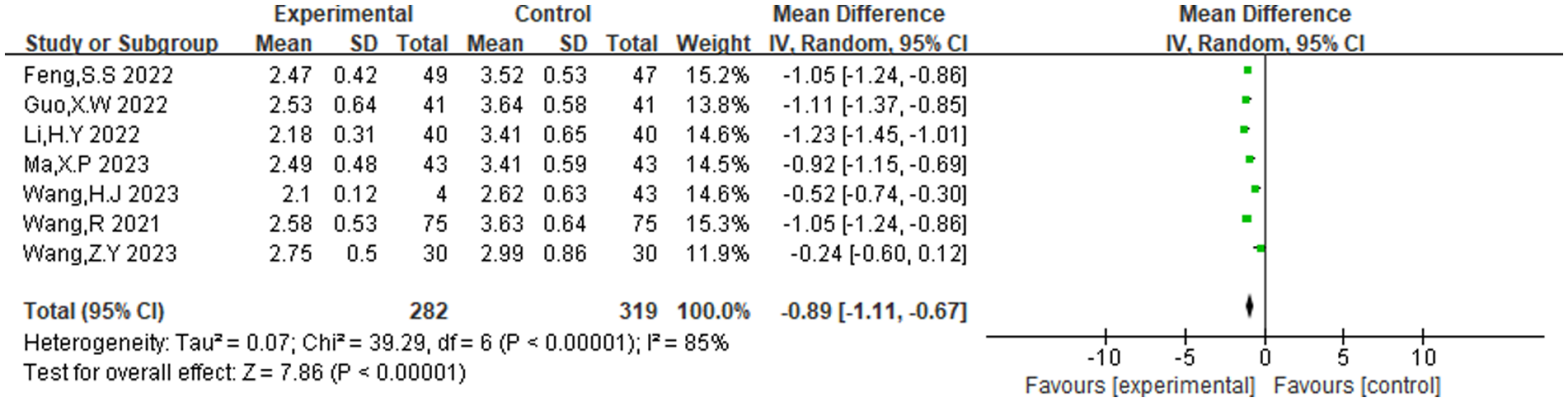
Meta-analysis of FIB in studies.

A total of 7 studies (13, 15, 22, 24, 25, 29, 30) reported FIB. After detecting heterogeneity (*p* < 0.00001, *I*^2^ = 85%), a random-effects model was adopted. The systematic review results demonstrated that the FIB level in the observation group was significantly lower than that in the control group, with a statistically significant difference (MD = −0.89, 95% CI [−1.11, −0.67], *p* < 0.00001), as shown in Figure 7.

To investigate the sources of high heterogeneity in FIB, we conducted a sensitivity analysis (refer to the Supplementary file). By sequentially removing each study, we observed that the range of the combined effect size (MD) varied between −0.83 and −0.98, with all results being statistically significant (*p* < 0.00001). Notably, the direction of the effect remained consistent with our primary analysis, indicating that no single study exerted a disproportionate influence on the overall effect. Nonetheless, the sensitivity analysis did not significantly reduce the heterogeneity, as evidenced by the *I*^2^ statistic remaining above 78%. This suggests that the heterogeneity may be attributed to clinical or methodological variations among the studies included in the analysis.

#### 3.3.7 CAL

**Figure 8:**
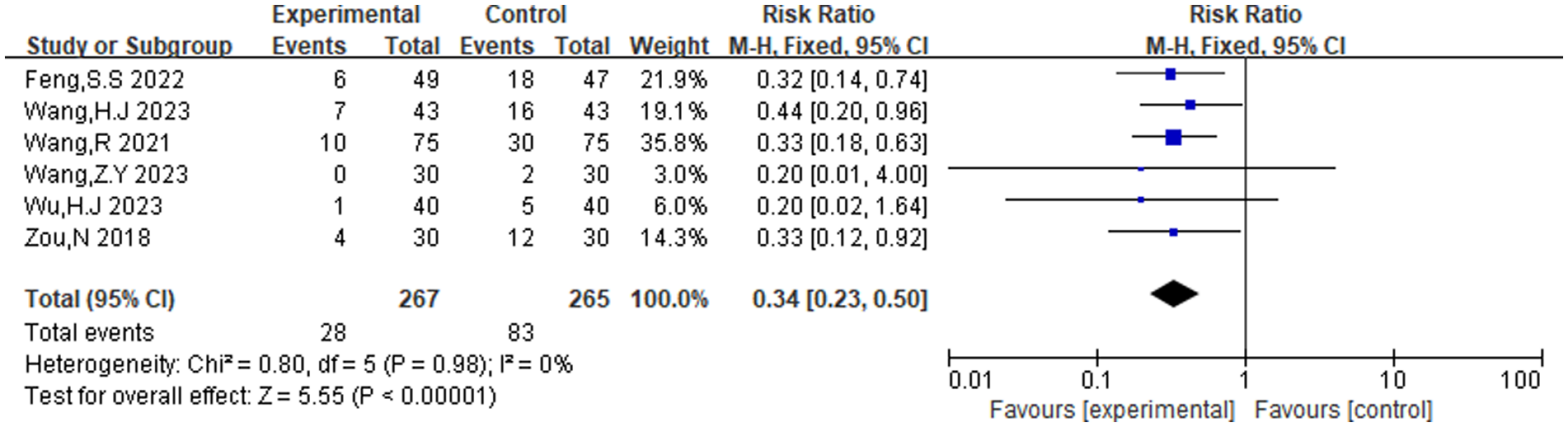
Meta-analysis of CAL in studies.

A total of 6 studies (13, 19, 22, 24, 25, 28) reported the incidence of CAL, and after detecting heterogeneity (*p* = 0.98, *I*^2^ = 0%), a fixed-effects model was employed. The results of the systematic review indicated that the incidence of CAL in the observation group was significantly lower than that in the control group, with a statistically significant difference (RR = 0.34, 95% CI [0.23, 0.50], *p* < 0.00001), as shown in Figure 8.

### 3.4 The adverse reactions

**Figure 9:**
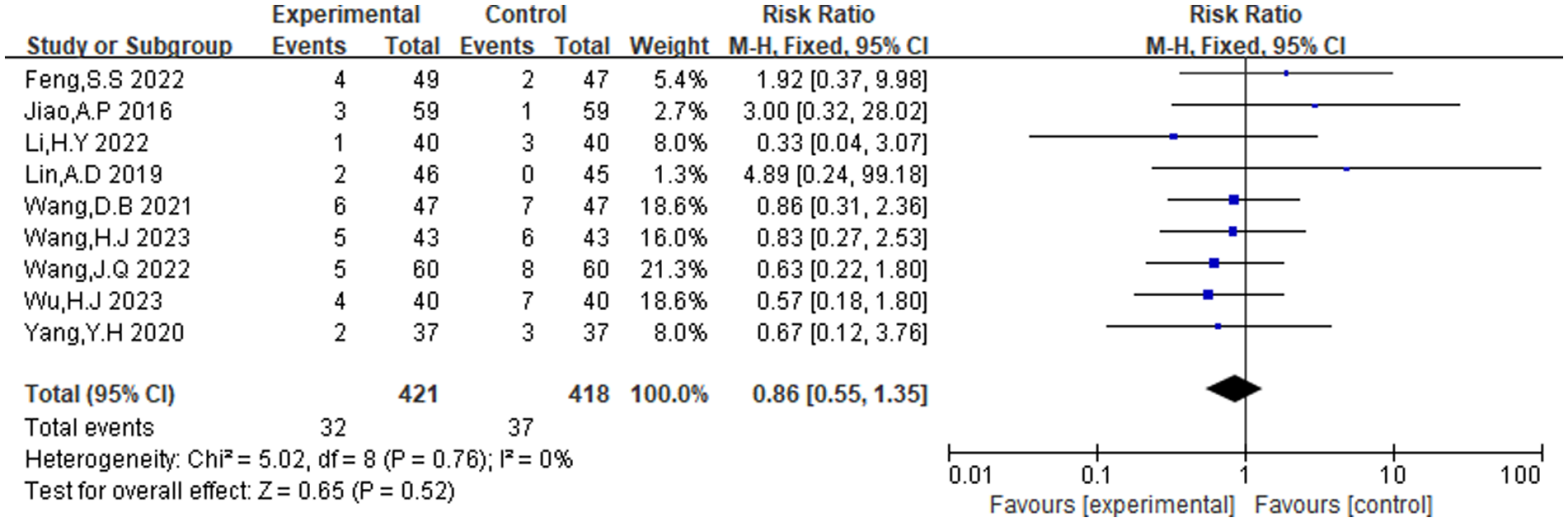
Meta-analysis of the incidence of adverse reaction in studies.

In recent years, the adverse reactions of combination drug regimens for the treatment of rare diseases in children have gradually become a focus of pediatric clinical practice. This article provides a detailed analysis of the adverse reactions associated with dual-drug combinations. A total of 9 studies (13, 15–17, 19, 21, 23, 25, 26) reported adverse reactions, primarily including dizziness, headache, nausea, vomiting, rash, and fever, as detailed in Table 4. A meta-analysis of adverse reactions between the two groups showed no statistically significant difference in the incidence of adverse reactions between the study group and the control group (RR = 0.86, 95% CI [0.55, 1.35], *p* = 0.52), as illustrated in Figure 9.

**Table 4:** Adverse reaction classification information (cases).

Literature source	Dizziness/headache		Nausea/vomiting		Rash		Fever	
	T	C	T	C	T	C	T	C
Feng et al. 2022 (13)	2	1	2	1	–	–	–	–
Li et al. 2022 (15)	–	–	1	3	–	–	–	–
Yang et al. 2020 (16)	–	1	1	1	1	1	–	–
Lin et al. 2019 (17)	2	–	–	–	–	–	–	–
Wu et al. 2023 (19)	1	2	3	5	–	–	–	–
Jiao et al. 2016 (21)	3	–	–	1	–	–	–	–
Wang et al. 2021 (23)	–	2	–	2	3	2	3	1
Wang et al. 2023 (25)	1	1	4	5	–	–	–	–
Wang et al. 2022 (26)	–	4	3	2	–	–	2	2
Total	9	11	14	20	4	3	5	3

### 3.5 Publication bias assessment

**Figure 10:**
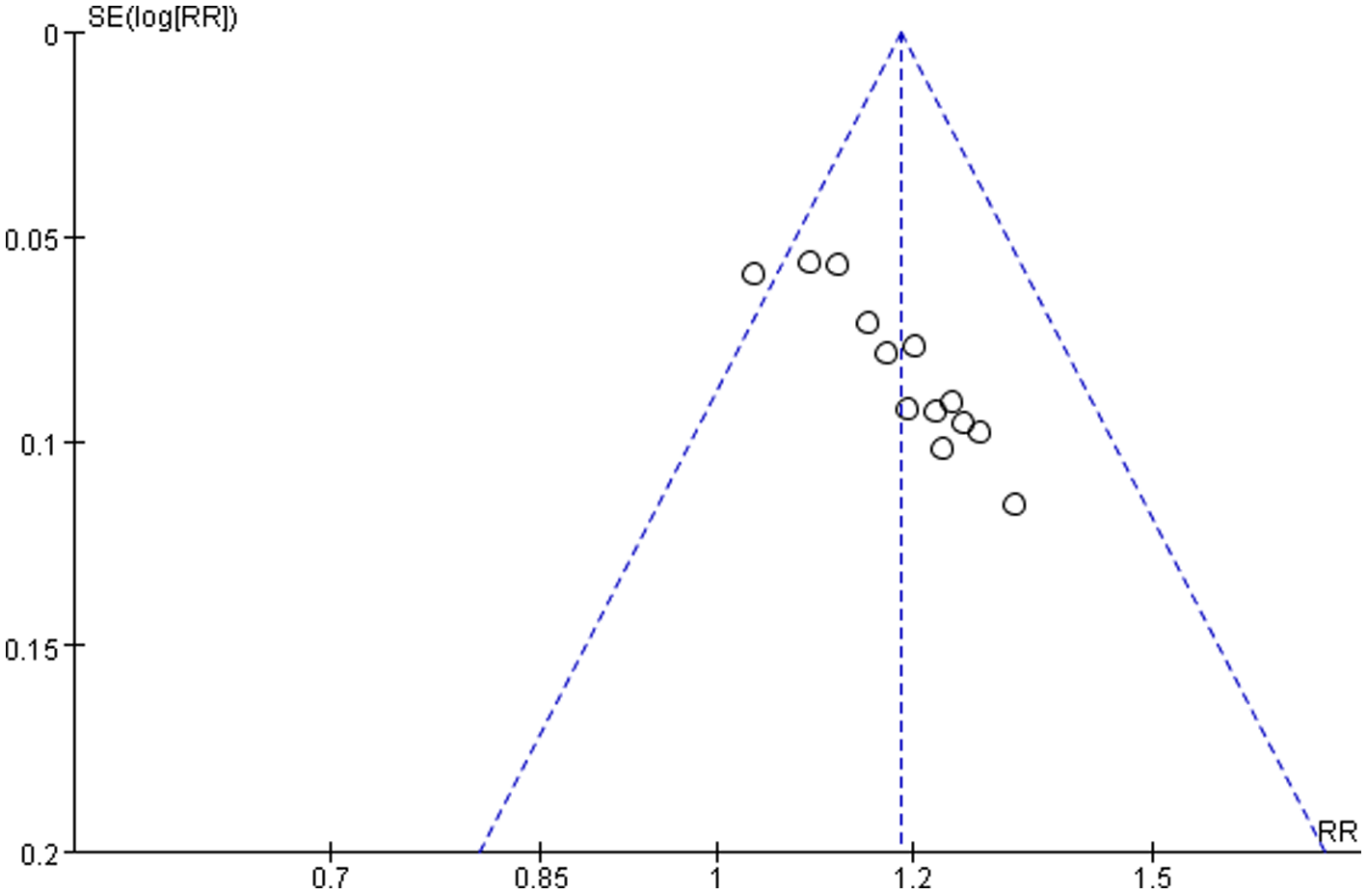
Funnel plot of total control rate of vomiting in two groups.

Figure 10 shows the funnel plot comparing the total effective rates of dipyridamole combined with immunoglobulin versus aspirin in treating Kawasaki disease in children. The 13 included studies are scattered on both sides of the funnel plot midline with some asymmetry, and one study falls outside the funnel plot, indicating potential publication bias in the study results.

## 4 Discussion

The combination of immunoglobulin and aspirin is the standard treatment regimen for pediatric Kawasaki disease. This regimen exhibits potent anti-inflammatory effects and can significantly reduce coronary artery damage in affected children. However, a considerable number of patients still develop immunoglobulin resistance or aspirin intolerance, which may exacerbate coronary lesions and even become life-threatening. In recent years, clinical research has been exploring novel therapeutic approaches for pediatric Kawasaki disease. Studies have found that dipyridamole can reduce vascular resistance, promote increased coronary blood flow, regulate hypercoagulable states, and improve coagulation function (31). Additionally, this medication can modulate immune function and mitigate inflammatory damage to coronary arteries. Consequently, dipyridamole has been gradually adopted in clinical practice for the treatment of pediatric Kawasaki disease. However, the Kawasaki disease diagnosis and treatment guidelines in China indicate that dipyridamole should not be used in children with severe coronary obstructive lesions, as it may lead to coronary “steal” phenomenon (manifested as vasodilatory adverse reactions such as dizziness and headache). However, there is no unified clinical standard for measuring severe coronary obstructive lesions, which also results in limited clinical application of dipyridamole.

This meta-analysis confirms the overall advantage of dual antiplatelet therapy in the treatment of Kawasaki disease in children. However, there are still some cases in clinical practice where the efficacy is suboptimal, indicating the presence of individual differences. With the development of precision medicine, pharmacogenomics provides a new perspective for achieving personalized treatment. For instance, in the case of psoriatic arthritis, studies have demonstrated that specific gene polymorphisms, such as those in the TNF-*α* gene, are closely related to patients’ responses to etanercept treatment. This suggests that genetic background can influence drug efficacy. Similarly, the treatment of Kawasaki disease may benefit from analogous pharmacogenomic studies. Future research could explore gene polymorphisms related to platelet function, immune regulation, and drug metabolism, thereby identifying subgroups of children who are most likely to benefit from dual antiplatelet therapy. This would facilitate the transition of treatment decisions from “population effectiveness” to “individual optimization,” enhancing the precision and safety of treatment. Pharmacogenomics holds great potential for achieving personalized treatment in Kawasaki disease, much like its demonstrated role in optimizing the use of biologics for conditions such as psoriatic arthritis. Murdaca et al. provided a valuable methodological framework through their research on the use of etanercept guided by pharmacogenomics (32, 33). Future studies could explore whether genetic markers can predict treatment responses or adverse reactions to dipyridamole in children with Kawasaki disease, ultimately guiding more personalized and effective treatment strategies.

The patient population included in this study comprised children both with and without baseline coronary artery damage. In future prospective studies, we aim to conduct stratified analyses based on baseline coronary status. This approach will enable a more precise evaluation of the independent role of dipyridamole in preventing new-onset coronary artery damage.

This study systematically evaluated the efficacy and safety of dipyridamole combined with immunoglobulin and aspirin in the treatment of Kawasaki disease in children. The results showed that the total effective rate in the observation group was higher than that in the control group, while the clinical symptom improvement time, CRP, ESR, PLT, CAL and FIB were all lower than those in the control group, with statistically significant differences (*p* < 0.05). Our research indicates that dipyridamole may be beneficial in alleviating inflammation and thrombosis in myocardial microvessels through its antiplatelet and anti-inflammatory effects. This finding provides a clear direction for the future design of more refined clinical studies, which should incorporate N proBNP measurements, cardiac ultrasound assessments, and detailed cellular phenotyping. The methodological limitations of this systematic review include: low quality of included literature, relatively small sample sizes, and wide variation in follow-up periods.

This study has several methodological limitations. First, all 18 RCTs are sourced from Chinese literature, and the implementation of blinding was not reported, which leads to an increased risk of measurement bias. Second, the methods for allocation concealment were not described, thereby heightening the potential for selection bias. Third, although the randomization methods mentioned the use of random number tables or random grouping, the specific processes for sequence generation and implementation were not detailed, making it challenging to evaluate the adequacy of randomization. Fourth, none of the included studies registered their clinical trials, which precludes the exclusion of publication bias and selective reporting bias. In conclusion, most outcome indicators demonstrated significant heterogeneity, and the sensitivity analyses did not reveal the sources of this heterogeneity. Due to the limited number of studies included, further investigation through subgroup analyses was not feasible. These deficiencies significantly diminish the evidence level of the conclusions drawn from this meta-analysis; thus, caution should be exercised when interpreting the results. Future studies should conduct multicenter, prospective, double-blind, registered RCTs to provide more reliable evidence-based support.

Since all RCTs included in this study were conducted in China, the generalizability and applicability of these results to other countries with differing healthcare systems, population characteristics, and medical resource conditions are limited. This limitation underscores the constraints of our research. International guidelines do not recommend dipyridamole as a routine first-line treatment for Kawasaki disease, leading to a scarcity of high-quality clinical studies on this topic. However, glucose-6-phosphate dehydrogenase deficiency is relatively common in China, and children with this condition are at risk of acute hemolysis when treated with aspirin. Consequently, combination therapy with dipyridamole has emerged as a clinical option for Kawasaki disease in East Asia. This choice not only reflects variations in medication availability but also highlights different strategies for managing the same disease across diverse healthcare environments.

## 5 Conclusion

Compared to the combined use of immunoglobulin and aspirin in the treatment of Kawasaki disease in children, the inclusion of dipyridamole may be associated with improved outcomes. However, further validation through high-quality, multi-center, double-blind randomized controlled trials is still necessary.

## Data Availability

The original contributions presented in the study are included in the article/Supplementary material, further inquiries can be directed to the corresponding author.
