# Supplementary material for: Dipyridamole combined with immunoglobulin and aspirin in the treatment of Kawasaki disease in children: a meta-analysis
Source: Front Med (Lausanne). 2026 Jun 29;13:1837742. doi: 10.3389/fmed.2026.1837742 (PMC13357125; doi:10.3389/fmed.2026.1837742)
Supplement: Supplementary file 1 [file Supplementary_file_1.docx]

Supplementary Material

# Supplementary Tables

**Supplementary Table 1.** Risk of bias summary for randomized studies.

| Study | Random sequence generation | Allocation concealment | Blinding participants/personnel | Blinding outcome assessment | Incomplete outcome data | Selective reporting | Other bias |
| --- | --- | --- | --- | --- | --- | --- | --- |
| Feng et al.2022^[13]^ | Low | Unclear | Unclear | Low | Low | Low | Unclear |
| Li et al. 2022^[14]^ | Low | Unclear | Unclear | Low | Low | Low | Unclear |
| Li et al. 2022^[15]^ | Low | Unclear | Unclear | Low | Low | Low | Unclear |
| Yang et al. 2020^[16]^ | Low | Unclear | Unclear | Low | Low | Low | Unclear |
| Lin et al. 2019^[17]^ | Low | Unclear | Unclear | Low | Low | Low | Unclear |
| Lin et al. 2022^[18]^ | Low | Unclear | Unclear | Low | Low | Low | Unclear |
| Wu et al. 2023^[19]^ | Low | Unclear | Unclear | Low | Low | Low | Unclear |
| Fu et al. 2022^[20]^ | Low | Unclear | Unclear | Low | Low | Low | Unclear |
| Jiao et al. 2016^[21]^ | Low | Unclear | Unclear | Low | Low | Low | Unclear |
| Wang et al. 2021^[22]^ | Low | Unclear | Unclear | Low | Low | Low | Unclear |
| Wang et al. 2021^[23]^ | Low | Unclear | Unclear | Low | Low | Low | Unclear |
| Wang et al. 2023^[24]^ | Low | Unclear | Unclear | Low | Low | Low | Unclear |
| Wang et al. 2023^[25]^ | Low | Unclear | Unclear | Low | Low | Low | Unclear |
| Wang et al. 2022^[26]^ | Low | Unclear | Unclear | Low | Low | Low | Unclear |
| Shao et al. 2022^[27]^ | Low | Unclear | Unclear | Low | Low | Low | Unclear |
| Zou et al. 2018^[28]^ | Low | Unclear | Unclear | Low | Low | Low | Unclear |
| Guo et al. 2022^[29]^ | Low | Unclear | Unclear | Low | Low | Low | Unclear |
| Ma et al. 2023^[30]^ | Low | Unclear | Unclear | Low | Low | Low | Unclear |

**Supplementary Table 2.** The result of sensitivity analysis.

| Outcome indicator | Exclusion of literature | Heterogeneity | MD（*95%*CI） | *P* |
| --- | --- | --- | --- | --- |
| CRP | Jiao,A.P 2016 | *P* < 0.00001, *I^2^* =97% | -9.61 (-12.93,-6.29) | *P* < 0.00001 |
|  | Li,H.Y 2022 | *P* < 0.00001, *I^2^* =98% | -10.50 (-14.33,-6.68) | *P* < 0.00001 |
|  | Lin,A.D 2019 | *P* < 0.00001, *I^2^* =98% | -9.67 (-13.10,-6.25) | *P* < 0.00001 |
|  | Lin,M.J 2022 | *P* < 0.00001, *I^2^* =98% | -9.86 (-13.36,-6.37) | *P* < 0.00001 |
|  | Shao,Q 2022 | *P* < 0.00001, *I^2^* =97% | -9.96 (-13.59,-6.32) | *P* < 0.00001 |
|  | Wang,H.J 2023 | *P* < 0.00001, *I^2^* =98% | -10.54 (-14.72,-6.37) | *P* < 0.00001 |
|  | Wang,J.Q 2022 | *P* < 0.00001, *I^2^* =96% | -11.47 (-14.34,-8.61) | *P* < 0.00001 |
|  | Wang,Z.Y 2023 | *P* < 0.00001, *I^2^* =98% | -11.60 (-14.96,-8.24) | *P* < 0.00001 |
|  | Wu,H.J 2023 | *P* < 0.00001, *I^2^* =98% | -10.15 (-13.65,-6.65) | *P* < 0.00001 |
|  | Yang,Y.H 2020 | *P* < 0.00001, *I^2^* =98% | -10.83 (-14.42,-7.24) | *P* < 0.00001 |
| ESR | Wang,H.J 2023 | *P* < 0.00001, *I^2^* =95% | -7.64 (-15.95,0.67) | *P* = 0.07 |
|  | Wang,J.Q 2022 | *P* =0.12, *I^2^* =53% | -9.70 (-12.66,-6.74) | *P* < 0.00001 |
|  | Wang,Z.Y 2023 | *P* < 0.00001, *I^2^* =96% | -7.04 (-12.78,-1.29) | *P* = 0.02 |
|  | Zou,N 2018 | *P* < 0.00001, *I^2^* =92% | -5.80 (-11.38,-0.22) | *P* = 0.04 |
| PLT | Wang,H.J 2023 | *P* =0.12, *I^2^* =83% | -49.18 (-101.26,2.90) | *P* = 0.06 |
|  | Wang,J.Q 2022 | *P* =0.04, *I^2^* =68% | -58.04 (-103.85,-12.23) | *P* = 0.01 |
|  | Wang,Z.Y 2023 | *P* =0.34, *I^2^* =7% | -18.61 (-31.70,-5.51) | *P* = 0.005 |
|  | Zou,N 2018 | *P* =0.002, *I^2^* =84% | -47.72 (-95.10,-0.33) | *P* = 0.05 |
| FIB | Feng,S.S 2022 | *P* < 0.00001, *I^2^* =87% | -0.86 (-1.13,-0.59) | *P* < 0.00001 |
|  | Guo,X.W 2022 | *P* < 0.00001, *I^2^* =87% | -0.85 (-1.11,-0.60) | *P* < 0.00001 |
|  | Li,H.Y 2022 | *P* < 0.00001, *I^2^* =84% | -0.83 (-1.07,-0.60) | *P* < 0.00001 |
|  | Ma,X.P 2023 | *P* < 0.00001, *I^2^* =87% | -0.88 (-1.15,-0.62) | *P* < 0.00001 |
|  | Wang,H.J 2023 | *P* =0.0003, *I^2^* =78% | -0.96 (-1.17,-0.76) | *P* < 0.00001 |
|  | Wang,R 2021 | *P* < 0.00001, *I^2^* =87% | -0.86 (-1.13,-0.59) | *P* < 0.00001 |
|  | Wang,Z.Y 2023 | *P* =0.0003, *I^2^* =79% | -0.98 (-1.17,-0.79) | *P* < 0.00001 |
| fever duration | Feng,S.S 2022 | *P* < 0.00001, *I^2^* =79% | -1.19 (-1.37,-1.00) | *P* < 0.00001 |
|  | Fu,J.L 2022 | *P* < 0.00001, *I^2^* =78% | -1.20 (-1.38,-1.01) | *P* < 0.00001 |
|  | Jiao,A.P 2016 | *P* < 0.00001, *I^2^* =78% | -1.20 (-1.38,-1.03) | *P* < 0.00001 |
|  | Li,W 2022 | *P* < 0.00001, *I^2^* =80% | -1.18 (-1.37,-1.00) | *P* < 0.00001 |
|  | Lin,A.D 2019 | *P* < 0.00001, *I^2^* =79% | -1.20 (-1.38,-1.02) | *P* < 0.00001 |
|  | Lin,M.J 2022 | *P* < 0.00001, *I^2^* =79% | -1.16 (-1.35,-0.98) | *P* < 0.00001 |
|  | Wang,D.B 2021 | *P* < 0.0001, *I^2^* =72% | -1.15 (-1.31,-0.98) | *P* < 0.00001 |
|  | Wang,H.J 2023 | *P* < 0.00001, *I^2^* =79% | -1.20 (-1.38,-1.02) | *P* < 0.00001 |
|  | Wang,R 2021 | *P* < 0.00001, *I^2^* =77% | -1.21 (-1.38,-1.03) | *P* < 0.00001 |
|  | Wang,Z.Y 2023 | *P* < 0.00001, *I^2^* =78% | -1.21 (-1.38,-1.03) | *P* < 0.00001 |
|  | Wu,H.J 2023 | *P* < 0.0001, *I^2^* =72% | -1.13 (-1.28,-0.98) | *P* < 0.00001 |
|  | Yang,Y.H 2020 | *P* < 0.00001, *I^2^* =79% | -1.20 (-1.38,-1.02) | *P* < 0.00001 |
|  | Zou,N 2018 | *P* < 0.00001, *I^2^* =77% | -1.15 (-1.33,-0.97) | *P* < 0.00001 |
| lymphadenopathy | Feng,S.S 2022 | *P* < 0.00001, *I^2^* =98% | -1.83 (-2.71,-0.96) | *P* < 0.0001 |
|  | Jiao,A.P 2016 | *P* < 0.00001, *I^2^* =98% | -1.82 (-2.66,-0.98) | *P* < 0.0001 |
|  | Li,W 2022 | *P* < 0.00001, *I^2^* =98% | -1.87 (-2.74,-1.01) | *P* < 0.0001 |
|  | Lin,A.D 2019 | *P* < 0.00001, *I^2^* =98% | -1.87 (-2.71,-1.03) | *P* < 0.0001 |
|  | Lin,M.J 2022 | *P* < 0.00001, *I^2^* =98% | -1.78 (-2.62,-0.94) | *P* < 0.0001 |
|  | Wang,D.B 2021 | *P* < 0.00001, *I^2^* =96% | -1.57 (-2.18,-0.97) | *P* < 0.00001 |
|  | Wang,H.J 2023 | *P* < 0.00001, *I^2^* =98% | -1.87 (-2.72,-1.02) | *P* < 0.0001 |
|  | Wang,R 2021 | *P* < 0.00001, *I^2^* =98% | -1.85 (-2.73,-0.97) | *P* < 0.0001 |
|  | Wu,H.J 2023 | *P* < 0.00001, *I^2^* =98% | -1.81 (-2.64,-0.99) | *P* < 0.0001 |
|  | Yang,Y.H 2020 | *P* < 0.00001, *I^2^* =98% | -1.82 (-2.65,-0.98) | *P* < 0.0001 |
|  | Zou,N 2018 | *P* < 0.00001, *I^2^* =98% | -1.58 (-2.32,-0.83) | *P* < 0.0001 |
| mucosal hyperemia | Feng,S.S 2022 | *P* < 0.00001, *I^2^* =82% | -1.87 (-2.21,-1.54) | *P* < 0.00001 |
|  | Fu,J.L 2022 | *P* < 0.00001, *I^2^* =81% | -1.78 (-2.11,-1.46) | *P* < 0.00001 |
|  | Jiao,A.P 2016 | *P* < 0.00001, *I^2^* =83% | -1.87 (-2.20,-1.54) | *P* < 0.00001 |
|  | Li,W 2022 | *P* < 0.00001, *I^2^* =81% | -1.87 (-2.22,-1.53) | *P* < 0.00001 |
|  | Lin,A.D 2019 | *P* < 0.00001, *I^2^* =83% | -1.85 (-2.18,-1.52) | *P* < 0.00001 |
|  | Lin,M.J 2022 | *P* < 0.00001, *I^2^* =82% | -1.79 (-2.11,-1.46) | *P* < 0.00001 |
|  | Wang,D.B 2021 | *P* < 0.00001, *I^2^* =76% | -1.74 (-2.03,-1.46) | *P* < 0.00001 |
|  | Wang,H.J 2023 | *P* < 0.00001, *I^2^* =83% | -1.86 (-2.19,-1.53) | *P* < 0.00001 |
|  | Wang,R 2021 | *P* < 0.00001, *I^2^* =82% | -1.87 (-2.21,-1.54) | *P* < 0.00001 |
|  | Wang,Z.Y 2023 | *P* < 0.00001, *I^2^* =82% | -1.88 (-2.21,-1.55) | *P* < 0.00001 |
|  | Wu,H.J 2023 | *P* < 0.00001, *I^2^* =83% | -1.81 (-2.13,-1.49) | *P* < 0.00001 |
|  | Zou,N 2018 | *P* < 0.00001, *I^2^* =79% | -1.75 (-2.06,-1.45) | *P* < 0.00001 |
